# ‘Get Your Life Back’: process and impact evaluation of an asthma social marketing campaign targeting older adults

**DOI:** 10.1186/1471-2458-13-759

**Published:** 2013-08-15

**Authors:** Uwana Evers, Sandra C Jones, Don Iverson, Peter Caputi

**Affiliations:** 1 Centre for Health Initiatives, University of Wollongong, Innovation Campus, ITAMS Building 233.G14, Squires Way, Fairy Meadow, Wollongong NSW 2522, Australia; 2 Faculty of Science, Medicine and Health, University of Wollongong, Wollongong, Australia; 3 School of Psychology and the Centre for Health Initiatives, University of Wollongong, Wollongong, Australia

**Keywords:** Process evaluation, Impact evaluation, Asthma, Older adults, Social marketing, Health promotion, Knowledge, Perceptions, Message recall, Materials recognition

## Abstract

**Background:**

Asthma in older adults is underdiagnosed and poorly self-managed. This population has little knowledge about the key symptoms, the prevalence among older adults, and the serious consequences of untreated asthma. The purpose of this study was to undertake a multifaceted evaluation of a social marketing campaign to increase asthma awareness among older adults in a regional Australian community.

**Methods:**

A cohort of older adults in an intervention region (*n* = 316) and a control region (*n* = 394) were surveyed immediately prior to and following the social marketing campaign. Campaign awareness, message recall, materials recognition, and actions taken as a result of the campaign were assessed in both regions. Asthma knowledge and perceptions, experience of asthma symptoms, and general health were also assessed in both regions at baseline and follow-up. Analyses were conducted to explore the effects of the campaign in the intervention region, and to examine outcomes among different audience segments.

**Results:**

The survey data showed that those in the target segments (*Wheezers* and *Strugglers*) had better message recall, and were more likely to report having taken action to control their respiratory symptoms. The campaign significantly increased the number of calls to an asthma information line from the target audience in the intervention community.

**Conclusions:**

A theory-based social marketing campaign conducted over 3-months increased the asthma information seeking behaviours of older adults in the intervention community compared to the control community. Recommendations are outlined for future community health promotion campaigns targeting older adults.

## Background

Research on asthma in older adults has increased considerably over the past few years [1-8] because the number of older adults with chronic disease is projected to rise as the population ages [9]. In Australia, the prevalence of asthma among individuals aged 55 years and over is approximately one in 10 [10], and the majority of deaths that are attributed to asthma occur in older adults [11]. Asthma tends to be more severe in older adults, and more so in those individuals who have had the disease for a long period [12]. Older adults with asthma are more likely to report poorer general health and lower quality of life than older adults without asthma [13,14], and have a heightened risk of premature disability and death [15]. There is a growing body of evidence to suggest that there are many older adults living with undiagnosed asthma [8,16-18]. Recent studies have demonstrated that, regardless of diagnosis, the experience of respiratory symptoms in older adults is related to poorer quality of life [19,20].

Older adults who have not received an asthma diagnosis tend to believe that asthma is primarily a disease that affects children, and do not perceive that they could be susceptible to developing the disease [21]. The experience of respiratory symptoms is often attributed to the normal ageing process [21,22], and those older adults with an asthma diagnosis often fail to properly self-manage their symptoms [8,14]. In addition, perceptions of symptom severity may deteriorate with age [23], some older adults may struggle to properly use their asthma medication [24], and some may not regard asthma as a chronic condition [6].

Recent reviews have recommended that governments and communities undertake public health programs to raise community awareness of asthma amongst older adults, enhance older adults’ understanding of asthma, and address commonly held misperceptions [5,25]. While there are several evidence-based frameworks available to structure health promotion interventions, the social marketing framework has been particularly effective in health promotion to increase knowledge of particular health issues and change targeted health behaviours [26-28]. Importantly, social marketing interventions have been shown to be successful in achieving health behaviour change among older adult populations [29-35].

### The development of an asthma awareness campaign for older adults

**Figure 1:**
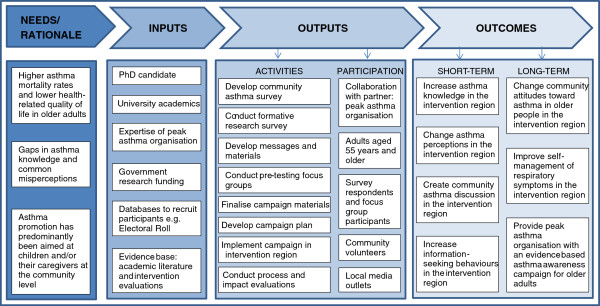
The logic model behind the ‘Get Your Life Back’ campaign.

A social marketing intervention was developed to increase community awareness of asthma among older adults, and to encourage those with respiratory symptoms to seek medical advice. The main aim of the intervention was to address the asthma misperceptions of older adults, highlighting that respiratory symptoms are not a normal part of ageing. The research followed the stages of social marketing: planning, message and materials development, pretesting, implementation, and evaluation [36]. The findings of the first three stages of the research are detailed elsewhere [20,37]. The current paper reports on the implementation and evaluation of the asthma social marketing campaign. Health communication and behaviour change theories were utilised to create a logic model for the campaign [38,39]. The logic model (see Figure 1) describes: the rationale behind the intervention; inputs, including resources available for the campaign; outputs, comprising of activities undertaken during the campaign, and participation of various stakeholders; and finally, target outcomes for the short-, medium-, and long-term as a result of the intervention.

Initial formative research was carried out through a large-scale, population-based survey of older adults (*n* = 4,066; response rate 46.8%) in the intervention and control regions [20]. In addition to questions on general health and asthma knowledge, the survey was based on the elements of the Health Belief Model [40]: perceptions of susceptibility to, and severity of, asthma; perceptions of benefits and barriers to visiting their doctor in relation to asthma symptoms; the cues that encourage individuals to visit their doctor; and their perceptions of self-efficacy in coping with the emotional and physical impacts of asthma symptoms. The data indicated that older adults generally perceived asthma to be a serious disease, although they tended to perceive that they were not highly susceptible to developing it [20]. Significant differences in general health ratings and perceptions of susceptibility were found between those who had recently experienced respiratory symptoms and those who had not; however, there were no significant differences in asthma knowledge, perceived self-efficacy, and perceived severity between those with and without an asthma diagnosis. Thus, audience segmentation was based on these two variables: recent experience of respiratory symptoms, and the presence or absence of an asthma diagnosis [20]. This created four distinct audience segments: *Wheezers*, *Breathers*, *Strugglers* and *Bloomers* (see Table 1). For example, *Strugglers* were those individuals who had recently experienced respiratory symptoms, but did not have an asthma diagnosis. The target groups for campaign messages were those with respiratory symptoms: *Wheezers* and *Strugglers*.

**Table 1:** Audience segmentation in the ‘Get Your Life Back’ campaign

**Segment**	**Sample size (*n*** = 3,757)*	**Proportion of population**	**Recent experience of respiratory symptoms**	**Ever received an asthma diagnosis**
**Wheezers**	540	14.4%	YES	YES
**Breathers**	112	3.0%	NO	YES
**Strugglers**	1,303	34.8%	YES	NO
**Bloomers**	1,792	47.8%	NO	NO

*Sample from initial formative research [20].

The campaign messages encouraged active self-management of respiratory symptoms so that breathing difficulties would no longer interfere with daily activities. Messages emphasised that difficulty in breathing is not a normal part of getting older. Specific messages for *Wheezers* and *Strugglers* were developed from survey data based on each segment’s distinct asthma knowledge and perceptions. *Wheezers* were encouraged to properly self-manage their asthma, and not ignore their symptoms. Messages for *Strugglers* focused on asthma prevalence among older adults and the key symptoms that, if present, could indicate asthma. Campaign materials directed older adults experiencing respiratory symptoms to contact an asthma information line or a website for more information on asthma, or to visit their doctor.

A design brief outlining the aims of the campaign, target audience, key messages, and formats for campaign materials was given to three groups of designers. Each of these groups created a set of campaign posters for testing with the target audience. Consequently, three different campaign concepts were pre-tested with four focus groups of older adults (*n* = 34) from the intervention region in order to determine the elements of campaign materials that would most effectively engage and inform older adults [37]. The findings suggested that older adults preferred images of ordinary older people undertaking everyday activities that they could relate to, and were more likely to engage with the materials when the campaign depicted a grandparent/grandchild relationship. Focus group participants responded positively to materials that compared two people with asthma to emphasise the positive impact of controlled asthma self-management on quality of life. Older adults were interested in learning new information about asthma prevalence and specific symptoms, preferred materials that asked questions like “Could it be asthma?” and “Can you spot the difference?”, and engaged most with the positive and empowering tagline “Get Your Life Back” [37]. The focus group findings directed the final design of the ‘Get Your Life Back’ campaign materials. The formative research phase ensured that the ‘Get Your Life Back’ campaign addressed the eight social marketing elements [41,42]. The application of the elements to the formation of ‘Get Your Life Back’ is described in Table 2.

**Table 2:** The eight social marketing elements in ‘Get Your Life Back’

**Social marketing element**	**Application to the ‘Get Your Life Back’ campaign**
**Consumer orientation**	Older adults from three regions were involved in various stages of the research from formative stages through to implementation and final evaluation. Researchers considered the health needs of the target audience, but more broadly concentrated on the determinants of quality of life in older adults. Formative research highlighted that being healthy to enjoy time with their grandchildren and family was highly valued, and could motivate older adults to take action and seek information.
**Insight**	It was important to gain insight into the asthma knowledge, perceptions, and health behaviours of older adults in our target communities. Over 4000 individuals from three regions responded to the initial large-scale formative research survey. The data demonstrated that almost half of the sample had recently experienced breathlessness, and that this experience was a predictor of lower mood and poorer health ratings [20]. Furthermore, this formative research gave insight into effective audience segmentation based on respiratory symptoms and asthma diagnosis. Subsequently, 34 older adults participated in focus groups to test campaign messages and materials [37]. This insight suggested that older adults respond best to positive and empowering health promotion materials that include individuals and everyday situations that they can personally relate to. Finally, more than 700 individuals from both intervention and control communities completed pre- and post-campaign surveys as part of the evaluation of the intervention.
**Segmentation**	The older adult population was segmented on the basis of two variables: recent experience of respiratory symptoms, and the presence of an asthma diagnosis. These variables created four segments: *Wheezers*, an asthma diagnosis and symptoms; *Breathers*, an asthma diagnosis, but no symptoms; *Strugglers*, no asthma diagnosis, but have symptoms; and *Bloomers*, no diagnosis and no symptoms. These segments had distinct differences in asthma knowledge and perceptions, general health ratings, and frequency of visits to the doctor. Due to their recent experience of respiratory symptoms, *Wheezers* and *Strugglers* were the main target of ‘Get Your Life Back’.
**Marketing mix**	*Product*. For individuals with respiratory symptoms, the product was the ability to undertake activities that would only be possible with improved respiratory health.
	*Price*. The possibility of receiving a diagnosis could cause concern for *Strugglers*; there may also have been a monetary cost for both target segments for medications needed to manage their respiratory symptoms.
	*Place*. Doctors and pharmacists, and the environments where they work, cafes, community and leisure centres, libraries, shopping centres frequently visited by older adults were the key places in this marketing mix.
	*Promotion*. In addition to advertising on bus shelters and in public bathrooms, postcards were delivered throughout the region. Promotion events were held in local shopping centres, and the campaign gained media attention on local TV and radio.
**Theory**	The Health Belief Model was used in conjunction with the social marketing framework to better understand the asthma perceptions of older adults and to determine how the campaign would best encourage website visits, calls to the information line, and visits to the doctor to discuss respiratory symptoms.
**Behaviour**	‘Get Your Life Back’ encouraged older adults with uncontrolled respiratory symptoms to seek further information about asthma on the internet or by telephone, and promoted the discussion of their respiratory symptoms with their doctor. In the future, this may lead to the adoption of appropriate self-management behaviours.
**Competition**	All other local and national health promotion interventions conducted in the same timeframe competed for older adults’ time and attention. Commercial advertising also competed with asthma awareness promotion efforts. ‘Get Your Life Back’ attempted to stand out from the competition by incorporating local images into the campaign materials, and by ensuring the placement of campaign materials was in locations visited regularly and frequently by older adults.
**Exchange**	‘Get Your Life Back’ highlighted that respiratory symptoms are not normal and encouraged older adults to take control of their health. The primary benefit offered to older adults with respiratory symptoms was the ability to participate more fully in the activities they enjoy by controlling the symptoms that may hinder their choices. In return, individuals had to be willing to give up their time to seek information; incur potential costs to visit the doctor, and potentially experience psychological distress in the instance of being diagnosed with asthma or another condition.

The primary objective of this paper is to describe the process and impact evaluations of the ‘Get Your Life Back’ social marketing campaign, which aimed to increase awareness of asthma in older adults and encourage action in those with respiratory symptoms. Four measurable aims follow from this objective: They are to: (1) evaluate the implementation fidelity of the campaign; (2) describe campaign awareness, message recall, and materials recognition; (3) measure change in asthma knowledge and perceptions; and (4) measure actions undertaken by older adults as a result of the campaign.

## Methods

This study utilised a pretest-posttest control group design [43]; data were collected from the same individuals before and after the campaign representing both the intervention and control regions. This design allowed changes in the intervention region to be compared with changes in the control region, and controlled for the main sources of internal invalidity [43]. The intervention and control regions were matched at baseline on demographic and socio-economic variables; any existing differences were controlled for in the analyses.

Surveys with older adults from the control and intervention regions were conducted in Summer 2012 (baseline) and again in Autumn 2012 (follow-up). Potential respondents were initially selected at random from the mandatory Australian Electoral Roll in an earlier phase of the research, and had then provided their details in order to participate in future stages of the asthma research. Surveys were sent to the 1,104 individuals who had registered their interest: 467 individuals from the intervention region and 637 from the control region. The chance to win a television was offered as an incentive to complete both baseline and follow-up surveys. In addition to collecting demographic information on the sample populations, the survey assessed baseline and follow-up measures of asthma knowledge and risk perceptions, and exposure to the asthma social marketing campaign messages and materials. Ten items were adapted from the Asthma Quality of Life Questionnaire [44]; these items assessed symptoms of breathlessness and mood over the previous four weeks. Two items assessed perceived general health, and perceived health compared to one year previous. Fifteen items were adapted from the Chicago Community Asthma Survey to examine asthma knowledge [45]. Two items assessed perceptions of asthma susceptibility and severity, and one item assessed whether the individual had ever received an asthma diagnosis from a doctor or nurse. In order to compare to the follow-up data after the intervention, respondents were also asked at baseline whether they had noticed any asthma promotion activities in their community; if they answered that they had seen asthma promotion, respondents were then asked the main messages they recalled, and to specify the locations where they had seen the promotional materials. The follow-up survey included all the baseline survey items, plus items on materials recognition and actions undertaken as a result of the asthma promotion activities. Ethics approval was obtained from the University of Wollongong’s Human Research Ethics Committee (HE11/433).

‘Get Your Life Back’ was implemented in several stages, beginning with the campaign launch in February 2012. A media brief was prepared and delivered to the six main local media outlets (one television station, two newspapers, and three radio stations) immediately prior to the campaign launch. The widely watched local television station aired a story in prime time on the launch of the ‘Get Your Life Back’ campaign. Two local radio stations also covered the launch.

The initial dissemination of posters and postcards took 12 volunteers approximately two weeks to reach 115 locations including community centres, pharmacies, cafes, leisure centres, and grocery stores across the region. Volunteers were given a briefing, and provided with detailed information about locations for delivery. In addition, materials were delivered to the local association of general practitioners for distribution to doctors’ waiting rooms and medical centres, and to librarians for placement within community libraries. Twenty bus shelter advertisements were placed along a busy free bus route in the city centre, and two large freestanding advertisements were placed in two shopping centres (four advertisements in total) for the duration of the campaign. Additionally, 47 advertisements with takeaway information cards were placed in public bathrooms in 10 clubs and shopping centres across the region. Tracking data was collected during the implementation phase to maintain a record of the number of materials distributed to each location. Volunteers who disseminated the materials recorded which materials were placed in each location, and whether particular locations refused participation.

Several strategies were implemented during the 3-month campaign period to maintain interest in the campaign. At the end of the first month, almost 90,000 campaign postcards were delivered to private letterboxes; one to every household in the region. There were two one-day stalls held in large shopping centres manned by researchers and volunteers to provide asthma information to the public. At the start of the third month, there was another media release on asthma.

The sources of evaluation data are summarised in Table 3. The process evaluation data were derived from campaign implementation records as well as the components of the baseline and follow-up surveys that assessed recall of asthma promotion. Data from the surveys provided impact evaluation measures of message recall, materials recognition, changes in asthma knowledge and perceptions, and actions undertaken as a result of the ‘Get Your Life Back’ campaign. In addition to the survey data, the peak asthma organisation provided de-identified databases with call data to determine the impact of the campaign on calls from older adults in the intervention region. Activity on the ‘Get Your Life Back’ campaign webpage was recorded with Google Analytics.

**Table 3:** Process and impact evaluation methods for ‘Get Your Life Back’

**Evaluation criteria**	**Measurement methods**
***Process evaluation***	
Implementation fidelity	Description of the extent to which the campaign was carried out as planned, including materials distribution and media exposure
Recall of asthma promotion	Baseline and follow-up questions about recall of asthma promotion in the community
Environmental factors	External factors that assisted and/or hindered the success of campaign implementation, including weather, competing news stories, and other interventions aimed at older adults
***Impact evaluation***	
Campaign recognition and message recall	Follow-up survey questions on asthma promotion recall, specific message recall, location of campaign materials, materials recognition, and perceptions of who were the target audience of the asthma messages
Change in asthma knowledge	Baseline and follow-up survey questions on asthma knowledge, including specific symptoms and triggers
Change in perceptions of susceptibility to and severity of asthma	Baseline and follow-up survey questions on perceptions of susceptibility to, and severity of, asthma
Calls to an asthma information line	Number of calls to the peak asthma organisation during the campaign, compared to periods immediately before and after, and compared to the same 3-month period the year before
Visits to the ‘Get Your Life Back’ campaign webpage	Number of website visits during the campaign period and length of time spent on the site
Visits to the doctor or other health professionals	Self-report from follow-up survey
Other behaviours resulting from the campaign	Self-reported discussions about asthma as a result of the campaign from the follow-up survey

## Results

### Implementation fidelity

Table 4 compares the planned campaign activities with the activities that actually eventuated during the implementa-tion of ‘Get Your Life Back’. There were 479 posters and approximately 9,000 postcards distributed in 115 locations across the region. In addition, 89,785 ‘Get Your Life Back’ postcards were delivered to all households in the region. Twenty bus shelter advertisements were placed along a bus route in the city centre, 47 advertisements in public bathrooms were displayed in 10 locations, and 1,170 takeaway cards were taken from the cardholders on the advertisements. There were nine news stories across local media channels during the three-month campaign period.

**Table 4:** Planned versus actual campaign activities

**Activity**	**Planned**	**Actual**
Posters in public areas	500 posters in	479 posters in
	126 locations	115 locations
Postcards in public areas	20,000 postcards	9,000 postcards
Get Your Life Back postcard delivery to households	90,000 postcards	89,785 postcards
Bus shelter advertisements	20 posters	20 posters
Advertisements in public bathrooms	47 posters in	47 posters in
	10 locations	10 locations
Takeaway business card advertisements in bathrooms	-	1,170 cards
Freestanding advertisements in shopping centres	4 posters	4 posters
Media exposure		
Television	Planned for as much exposure as possible; Especially at the launch	1 news story
Radio		4 stories
Newspapers		2 stories
Internet		2 stories
Manned stalls in shopping centres	2 days	2 days
Newsletters	Any relevant	2 newsletters

### Baseline and follow-up surveys

Seven hundred and ten respondents aged 55 years and older completed both baseline and follow-up surveys (Table 5); this sample equated to a 66.4% response rate, accounting for “Returned to Senders” and ineligible responses. There were 316 respondents from the intervention region, and 394 from the control region. A higher proportion of female respondents (57.3%) than expected completed the surveys. The average age of respondents was 67.7 years (*SD* = 8.3); ages ranged from 55 to 95 years. Almost one-fifth (18.6%) responded that they had been diagnosed with asthma, which is comparable to other older adult samples e.g. [46]. Differences in campaign awareness, message recall, materials recognition, and actions undertaken as a result of the campaign were measured using McNemar’s and chi-square tests. Changes in asthma knowledge and perceptions were examined using two-way mixed design analysis of variance (ANOVA). ANOVAs examined differences between groups for: the intervention and control region; those who reported seeing asthma promotion activities and those who did not; and the four audience segments.

**Table 5:** Descriptive statistics of respondent demographics

	**Baseline (pre-campaign)**			**Follow-up (post-campaign)**		
	***n *(%)**			***n *(%)**		
**Region**	**Intervention**	**Control**	**TOTAL**	**Intervention**	**Control**	**TOTAL**
**Respondents**	357 (44.2)	451 (55.8)	808 (100)	316 (44.5)	394 (55.5)	710 (100)
**Sex**						
Male	167 (46.8)	185 (41.0)	352 (43.6)	143 (45.3)	160 (40.6)	303 (42.7)
Female	190 (53.2)	266 (59.0)	456 (56.4)	173 (54.7)	234 (59.4)	407 (57.3)
**Age**						
Mean	67.0	67.6	67.3	67.5	67.9	67.7
SD	8.0	8.7	8.4	7.9	8.6	8.3
Range	55-88	55-96	55-96	55-89	55-95	55-95
**Cultural diversity**						
Born overseas	103 (28.9)*	54 (12.0)*	157 (19.4)	92 (29.1)*	45 (11.4)*	131 (15.5)
Other language	19 (5.3)	16 (3.6)	35 (4.3)	17 (5.4)	11 (2.8)	28 (3.9)
ATSI	3 (0.8)	4 (0.9)	7 (0.9)	3 (1.0)	3 (0.8)	6 (0.9)
**Asthma diagnosis**						
**(ever)**	71 (19.9)	98 (21.7)	169 (20.9)	56 (17.7)	76 (19.3)	132 (18.6)

Chi-square differences between regions * *p* < .001.

The proportion of each segment represented in the follow-up sample differed from the wider target audience population, χ^2^ (3, *N* = 696) = 8.77, *p* = .032 (see Table 1 & Table 6). The target segments, *Wheezers* (13.8%) and *Strugglers* (33.5%) accounted for almost half (47.3%) of the sample; this proportion is slightly less than the expected 49.2% from the formative research sample of older adults [20].

**Table 6:** Descriptive statistics of the audience segments

**SEGMENTS**	**Pre-campaign survey (*n*** **= 808)**		**Post-campaign survey (*n*** **= 710)**	
	*n*	%	*n*	%
**Wheezers**	119	15.0	96	13.8
**Breathers**	48	6.0	34	4.9
**Strugglers**	268	33.7	233	33.5
**Bloomers**	360	45.3	333	47.8
*Missing*	*13*	*0*	*14*	*0*

### Campaign awareness

Almost one-fifth (18.5%; *n* = 58) of respondents in the intervention region stated that they saw asthma promotion materials during the campaign period. McNemar’s tests were conducted to examine whether the number of individuals who saw asthma promotion activities increased from pre- to post-test, in both the intervention and control regions. The test showed a significant increase in campaign awareness in the intervention region (*p* < .001); a significantly larger proportion of respondents reported that they had seen asthma promotion in their community at post-test (18.4%; *n* = 57) compared to pre-test (7.8%; *n* = 24). Comparatively, there was no increase in the control region in respondents reporting having seen asthma promotion from pre-test (11.1%; *n* = 43) to post-test (13.2%; *n* = 51) (*p* = .322). Across both communities, a significantly greater proportion of *Wheezers* (25.8%; *n* = 25) noticed asthma messages in their communities than any other segment (*Breathers*: 12.2%, *n* = 5; *Strugglers*: 12.6%, *n* = 29; *Bloomers*: 15.0%, *n* = 49), χ^2^ (3, *N* = 695) = 9.72, *p* = .021.

### Message recall

Individuals in the intervention region who reported seeing the campaign (*n* = 58) recalled messages to differing extents; almost half (47.4%; *n* = 27) recalled that “Shortness of breath is not a normal part of getting older”, and almost a quarter (24.6%; *n* = 14) recalled that “1 in 10 older Australians have asthma”. Some individuals recalled the campaign slogans “Get Your Life Back” (50.9%; *n* = 29) and “Could it be asthma?” (35.1%; *n* = 20). Only small proportions recalled our decoy messages: 5.3% (*n* = 3) recalled that “You can only get asthma as an adult if you had it as a child” and 3.5% (*n* = 2) responded that they heard “More men get asthma than women”. The proportion in the intervention region (50.9%; *n* = 29) that recalled the “Get Your Life Back” message was significantly higher than the control region (31.4%; *n* = 16), χ^2^ (1, *N* = 108) = 4.21, *p* = .040. Greater proportions of the two segments of individuals with an asthma diagnosis (*Wheezers*: 42.4% and *Breathers*: 50.0%) recalled the message that one in ten older Australians have asthma than the other two segments (*Strugglers*: 17.4% and *Bloomers*: 14.5%), χ^2^ (3, *N* = 145) = 12.10, *p* = .007.

### Materials recognition

The proportion of respondents in the intervention region that recalled seeing the “This is not a two-man job” poster, χ^2^(1, *N* = 156) = 13.92, *p* < .000, the “This is not Mount Everest” poster, χ^2^(1, *N* = 154) = 9.93, *p* = .002, and the Get Your Life Back postcard delivered to every household throughout the intervention region, χ^2^(1, *N* = 154) = 4.44, *p* = .035, was significantly higher than in the control region. There were no significant differences in materials recognition of the other posters between the two regions.

### Location of messages and materials

A significantly higher proportion of respondents in the intervention region recalled seeing asthma messages on bus shelters (12.3% vs. 2.0%), χ^2^ (1, *N* = 108) = 4.18, *p* = .041 and in other locations (for example, in public bathrooms and on television) (32.1% vs. 16.0%), χ^2^ (1, *N* = 108) = 3.72, *p* = .054, than the control region. The proportion of respondents in the control region that saw asthma messages in pharmacies (51.0% vs. 21.1%), χ^2^ (1, *N* = 108) = 10.57, *p* = .001, and doctors’ surgeries or medical centres (74.5% vs. 49.1%), χ^2^ (1, N = 108) = 7.30, *p* = .007, was significantly higher than the intervention region.

### Asthma perceptions

Results indicated that there were no changes in asthma perceptions as a result of the ‘Get Your Life Back’ campaign. Two-way mixed design ANOVAs were used to compare perceptions of susceptibility to, and severity of, asthma between the intervention and control regions before and after the asthma intervention. Results indicated that there was no significant change in perceptions of susceptibility to asthma, *F*(1,689) = 0.638, *p* = .425, or in perceptions of asthma severity, *F*(1,690) = 0.054, *p* = .817, following the intervention in either region. Perceptions of susceptibility to asthma, *F*(1,309) = 0.864, *p* = .353, and perceptions of asthma severity, *F*(1,309) = 0.227, *p* = .634, did not change significantly among those who had seen asthma promotion activities compared to those that had not seen any asthma promotion in the intervention region. There were also no differences between the segments from pre- to post-test in asthma susceptibility perceptions, *F*(3,678) = 0.619, *p* = .277, or asthma severity perceptions, *F*(3,679) = 0.456, *p* = .713.

### Asthma knowledge

There were no changes in asthma knowledge from pre- to post-test. Two-way mixed design ANOVAs were used to compare knowledge of asthma symptoms and triggers between the intervention and control regions before and after the asthma intervention. Results indicated that there was no significant change in the knowledge of asthma symptoms, *F*(1,676) = 2.928, *p* = .088, or asthma triggers, *F*(1,678) = 0.962, *p* = .327, following the intervention in either region. Knowledge that specific symptoms indicated asthma did not increase over time in either region: shortness of breath, *F*(1,686) = 1.604, *p* = .206; tightness in the chest, *F*(1,680) = 1.411, *p* = .235; and wheezing after exercise, *F*(1,682) = 1.914, *p* = .167. Knowledge that a cough at night could indicate asthma increased slightly, but significantly, in the control region, *F*(1,680) = 3.993, *p* = .046. There were no differences in knowledge of asthma symptoms, *F*(1,304) = 0.030, *p* = .863, or triggers, *F*(1,306) = 0.053, *p* = .818, between those who had and had not seen asthma promotion activities in the intervention region from pre- to post-test. There were no segment differences in the knowledge of symptoms, *F*(3,667) = 0.884, *p* = .449, or triggers, *F*(3,669) = 0.403, *p* = .751, following the intervention. Significant differences in knowledge levels were found when testing these groups at post-test only; the symptom knowledge of those who reported seeing asthma promotion activity in the intervention region was significantly higher than those who did not see any asthma promotion, *F*(1,309) = 19.813, *p* = .001, and the trigger knowledge of those who had seen asthma promotion activities was also higher than those who had not seen any promotional activities, *F*(1,309) = 11.828, *p* = .010.

### Actions undertaken as a result of the campaign

The number of calls to an asthma information line from older adults in the intervention region increased during the campaign, compared to the same period the year before, the three-month period before and the three-month period following the campaign (Table 7). The proportion of calls from older adults also increased during the campaign period. Statistical analyses on these data were not possible due to the small number of calls.

**Table 7:** Summary of calls to the asthma information line

**Calls**	**One year prior**	**Period before**	**CAMPAIGN**	**Period after**
Intervention region	8	9	29	13
Older adults (50 yrs+)	4	2	17	2
Diagnosis	2	2	6	2
No diagnosis	0	0	6	0
Unknown	2	0	5	0

There were 76 unique visits to the campaign webpage, 180 visits in total, during the campaign. The average time spent on the webpage was 47 seconds, compared to the average of 32 seconds across the entire site of the peak asthma organisation.

The audience segments differed significantly in their actions following the campaign. The target segments, *Wheezers* and *Strugglers*, reported a visit to a health professional significantly more than the non-target segments, χ^2^(1, *N* = 145) = 15.19, *p* < .001, while the two non-target segments, *Breathers* and *Bloomers*, reported taking no action significantly more than the target segments, χ^2^(1, *N* = 145) = 6.91, *p* = .009. Specifically, a greater proportion of *Wheezers* (27.3%) and to a lesser extent *Strugglers* (14.0%) reported seeing a health professional as a result of the campaign, compared to no *Bloomers* or *Breathers*, χ^2^(3, *N* = 145) = 18.76, *p* < .001.

## Discussion

To our knowledge, this is the first study to develop, implement and evaluate a theory-based social marketing campaign to address asthma knowledge and perceptions in older adults. The intervention had a modest impact in the target community; this occurred despite the short duration of the campaign.

The majority of campaign activities were carried out as planned. While there were locations, including some cafes and pharmacies, that declined to participate in the campaign, other unplanned locations were added to the distribution list during implementation. The number of postcards required for the campaign was overestimated; thus more than half of the postcards remained undistributed. The takeaway information cards alongside the bathroom advertisements were unplanned, so there was no estimation of the number that would be taken. The plan for the campaign was to elicit as much local media coverage as possible for the launch, and then sporadically during the campaign to maintain the interest of older adults. The campaign launch gained local television and radio coverage, although it was difficult to attract media interest during the campaign. Media coverage at different points in the campaign was connected with spikes in activity on the campaign webpage and calls to the asthma information line.

### Campaign awareness, message recall and materials recognition

The proportion of respondents in the intervention region that reported seeing the ‘Get Your Life Back’ campaign (18.5%) was comparable to other community-level cam-paigns targeting older adults: 17.4%-19.4% (*n* = 135–150) recalled a healthy eating and physical activity campaign in Alberta, Canada [47]; and 25% (*n* = 34) recalled seeing chronic obstructive pulmonary disease campaign posters in Salford, UK [48]. National campaigns and those that utilised advertising in mass media gained higher levels of exposure and consequently, higher levels of campaign awareness, than ‘Get Your Life Back’: recall of a US diabetes campaign ranged from 30% of the general public to 58% of those with diabetes [49]; in Alberta, Canada, recall of a back pain campaign increased from 31.9%-49.2% over the three years of the campaign [50]; and recall of a four-year physical activity campaign in New Zealand increased from 30% to 57% during the campaign [51]. As expected, a greater proportion of individuals in the intervention region responded that they recalled asthma promotion in their community compared to the control region.

Furthermore, the segmentation utilised in ‘Get Your Life Back’ was successful in identifying and reaching particular older adults; *Wheezers* noticed campaign materials significantly more than the other three segments. Not surprisingly, the main slogan of the campaign, ‘Get Your Life Back’, was recalled significantly more by those in the intervention region than in the control region. In terms of location, campaign materials were more likely to be recognised in the intervention region in specific locations such as bus shelters and in public bathrooms. Existing asthma promotion in the control region meant that recognition of promotional materials in pharmacies and medical centres was unexpectedly higher than in the intervention region.

### Asthma perceptions and knowledge

There were no significant changes in asthma perceptions or knowledge in the intervention community from pre- to post-test. The increase in knowledge of asthma symptoms in the control community at post-test was negligible, corresponding to the same average number of correct answers at both baseline and follow-up, and there was no significant change in knowledge of asthma triggers. At post-test in the intervention region, the asthma knowledge of symptoms and triggers of those who had seen the campaign was significantly higher than those who did not recall seeing any asthma promotion. However, this result could indicate that individuals with higher knowledge are more likely to notice asthma promotion messages, rather than the direct impact of campaign activities.

The control region had existing asthma outreach programs conducted by the state peak asthma organisation targeting other groups. These programs had been running since 2003, and included: one hour face-to-face training for school staff, covering the signs and symptoms of asthma, medications, asthma management in the school environment, and asthma first aid; nationally certified three hour face-to-face Emergency Asthma Management training to childcare staff to address the above aspects in greater depth; and asthma hardcopy resource provision to health professional settings such as hospitals and doctors’ clinics upon request. While these programs did not directly target older adults, individuals over 55 years could have been exposed to these activities, which could have contributed to the increase in asthma knowledge in the control region. In the same period, the intervention region received regular education sessions from peak asthma organisations directed at school staff, staff at aged care facilities, and community groups. These sessions were part of a state-wide asthma management program, and were also received by parts of the control region.

### Actions as a result of the campaign

The behavioural outcomes of the campaign give support to the segmentation of the older adult audience by recent experience of respiratory symptoms and asthma diagnosis; the target segments were more likely to take action and visit a health professional, while the non-target segments were more likely to take no action. There were also notable differences between the two target segments: *Wheezers* were more likely to take action than *Strugglers*, as they already had an asthma diagnosis. Furthermore, *Wheezers* may have perceived asthma promotion materials as more relevant, and may therefore be more likely to make attempts to take control of their respiratory symptoms. Importantly, almost a quarter of the *Wheezers* and more than 10% of *Strugglers* who saw ‘Get Your Life Back’ reported visiting their doctor as a result of seeing the campaign.

Most webpage activity and calls to the information line occurred in the two weeks following the campaign launch, and then tapered off over time. Similar to another community intervention [52], there were spikes in calls and website hits when the campaign attracted media attention. However, the initial level of interest and campaign activity was difficult to maintain due to lack of human resources and budget constraints (i.e. unable to produce advertisements for local television and radio stations).

### Strengths and limitations

A key strength of this study was the longitudinal design of the evaluation; this design allowed for a comparison between the intervention region and a control region, and enabled an assessment of the effects of the campaign. In addition, all elements of social marketing were considered in the development of the ‘Get Your Life Back’ campaign, unlike many interventions cited in reviews of social marketing campaign effectiveness [27,28]. Segmentation of older adults on the basis of asthma diagnosis and recent experience of breathlessness appeared effective, as there were significant differences in the outcomes for the different segments. *Wheezers* were the most likely to take notice of campaign activities, and both target segments – *Wheezers* and *Strugglers* – were more likely to visit a health professional as a result of engaging with ‘Get Your Life Back’. The non-target segments of older adults – *Breathers* and *Bloomers* – were more likely to report taking no action after seeing the campaign. Another strength of the campaign was the use of community volunteers; they were well utilised during the implementation period following the campaign launch. Through word of mouth, and expressions of interest during earlier phases of research, community members were recruited to disseminate the campaign materials around the intervention region.

There were limitations worth noting. First, the scales utilised to measure asthma knowledge and perceptions may not have been sensitive enough to detect small changes. Perceptions of susceptibility and severity were each examined by a single item, while the adapted asthma knowledge scale may not have detected differences because average scores were quite high at baseline, thereby creating a ceiling effect. More likely however, the lack of significant change in asthma perceptions and knowledge could be explained by limited exposure of older adults to ‘Get Your Life Back’. Modest exposure of the campaign to older adults largely resulted from the limited use of mass media and the short duration of the campaign. The utilisation of mass media channels such as television and radio over an extended time would increase the frequency of message delivery and, consequently, the opportunity for older adults to engage with the campaign and take action. Further, due to the short duration and resource constraints, there was no audit of the poster and postcard locations during the three months to check that the materials were still visible. Anecdotally, two organisations involved with the campaign may not have disseminated the materials as specified in the plan. In terms of the comparison between regions, the evaluation could not account for the effects of existing asthma programs.

## Conclusion

A community-level social marketing campaign was implemented to encourage older adults with recent respiratory symptoms to visit their doctor, call a dedicated asthma information line, and/or visit a campaign website for more asthma information. While the campaign was relatively small in terms of duration and resources, it resulted in some behaviour change in the target audience. ‘Get Your Life Back’ effectively increased calls to an asthma information line, and prompted older adults who had recently experienced respiratory symptoms to visit their doctor. The campaign may have initiated greater levels of information seeking behaviours among older adults had the campaign been carried out for a longer period of time, and utilised local mass media outlets such as radio and television as part of the advertising strategy.

Six key recommendations are made on the basis of the ‘Get Your Life Back’ evaluation (Table 8). Health promoters interested in the development of asthma awareness campaigns targeting older adults should carefully consider: the segmentation of their target audience, the planned duration of campaign activities, the number of resources necessary to adequately audit campaign implementation, strategies to raise awareness among health professionals in addition to the target audience, the use of community partnerships to strengthen the impacts of the campaign, and the utilisation of mass media to ensure maximum exposure of older adults to campaign messages. Effective interventions to increase asthma awareness and improve self-management of respiratory symptoms in older adults could result from the incorporation of these specific strategies in addition to the utilisation of social marketing techniques.

**Table 8:** Lessons learned from ‘Get Your Life Back’

**Recommendations for asthma awareness interventions to older adults**	
1.	Segment the audience on the basis of experience of breathlessness and asthma diagnosis
2.	Plan for the campaign duration to be longer than three months in order to maximise engagement with target segments
3.	Audit the locations of the campaign materials during the intervention period to ensure messages reach older adults
4.	Raise awareness of the campaign among health professionals that are directly impacted by the intended behavioural outcomes for older adults
5.	Form partnerships within the community, and utilise community volunteers
6.	Utilise wide-reaching forms of mass media (e.g. television and radio), both paid and unpaid, to maximise campaign reach and exposure to target segments

A small-scale asthma awareness social marketing campaign targeting older adults in a regional Australian community increased the number of calls to an asthma information line and initiated visits to a campaign website. After just three months, the campaign achieved levels of recognition comparable to other interventions of a similar size and scope [47,48]. Importantly, the target segments engaged with ‘Get Your Life Back’, and were more likely to take action as a result of the campaign compared to the non-target audience segments. This was the first study to demonstrate the effectiveness of this segmentation strategy in the promotion of respiratory symptom control to older adults. The ‘Get Your Life Back’ campaign could be modified, tailored to the specific needs of other older adult audiences, and conducted in communities around Australia. The intervention could also be expanded and provided on a wider-scale at the state or national level. In addition, new formative research could be undertaken to modify the intervention for application to other chronic diseases such as diabetes, hypertension, and arthritis in order to address misperceptions, increase knowledge and initiate information-seeking behaviours. Though ‘Get Your Life Back’ achieved only modest changes in asthma knowledge in the intervention region, future interventions that ensure more frequent exposure to relevant campaign messages over an extended period of time could expect to observe changes in knowledge and perceptions of a particular health issue. These changes in knowledge and perceptions should lead to changes in health behaviours and, in the longer-term, improved health outcomes for older adults. Future health promotion activities directed at older adults should apply the recommendations from this evaluation, and extend the findings of this research to other chronic diseases and other communities.

### Consent

Focus group participants and survey respondents were made aware that their data would be used in publications and reports. Written informed consent was obtained from focus group participants. Informed consent from survey respondents was implied by the return of the surveys.

## Competing interests

The authors declare that they have no competing interests.

## Authors’ contributions

UE contributed to the design of the study, carried out the data collection and analysis, and drafted the manuscript. SJ and DI contributed to the design of the study and provided feedback on the manuscript. PC provided statistical guidance and feedback on the manuscript. All authors read and approved the final manuscript.

## Pre-publication history

The pre-publication history for this paper can be accessed here:

http://www.biomedcentral.com/1471-2458/13/759/prepub
